# Formulation and Evaluation of *in situ* Gels Containing Clotrimazole for Oral Candidiasis

**DOI:** 10.4103/0250-474X.57291

**Published:** 2009

**Authors:** N. M. Harish, P. Prabhu, R. N. Charyulu, M. A. Gulzar, E. V. S. Subrahmanyam

**Affiliations:** NGSM Institute of Pharmaceutical Sciences, Paneer, Deralakatte Post, Mangalore-574 160, India

**Keywords:** Mucoadhesive *in situ* gels, prolonged release, carbopol, gellan gum, hydroxypropylmethylcellulose, clotrimazole

## Abstract

Gel dosage forms are successfully used as drug delivery systems to control drug release and protect the medicaments from a hostile environment. The main objective is to formulate and evaluate *in situ* oral topical gels of clotrimazole based on the concept of pH triggered and ion activated systems. The system utilizes polymers that exhibit sol-to-gel phase transition due to change in specific physico-chemical parameters. A pH triggered system consisting of carbopol 934P (0.2-1.4% w/v) and ion triggered system using gellan gum (0.1-0.75% w/v) along with hydroxylpropylmethylcelluose E50LV was used to prolong the release of clotrimazole (0.1% w/v). Formulations were evaluated for gelling capacity, viscosity, gel strength, bioadhesive force, spreadability, microbiological studies and *in vitro* release. The use of carbopol as *in situ* gel forming system was substantiated by the property to transform into stiff gels when the pH was raised, whereas in gellan gum this transformation occurred in the presence of monovalent/divalent cations. Effect of calcium carbonate and other process parameters optimized and found that increase in calcium ions produced stronger gels. The drug content, clarity, and pH of the formulation were found to be satisfactory. The viscosity was found to be in the range 5 to 85 centipoise for the sol, whereas for the gels it was up to 16000 centipoise. The formulation showed pseudoplastic flow with thixotrophy. The maximum gel strength (using texture analyzer) and bioadhesion was found to be up to 6.5 g and 4 g, respectively. The optimized formulations were able to release the drug up to 6 h. The formulation containing gellan gum showed better sustained release compared to carbopol based gels.

Oral candidiasis is one of the most common pathological conditions affecting the oral mucosa. Local delivery of drugs to the tissues of the oral cavity has a number of applications including the treatment of toothache, periodontal diseases, dental caries, bacterial and fungal infections. The conventional formulations for the local delivery of drugs to the oral cavity are the mouth paints, rinses, troches, creams and suspensions[1,2]. The reason for incomplete eradication of candidiasis in most cases may be due to the short residence time of antifungal agents in the oral cavity. The other reason may be degradation of antifungal agents in salivary fluid. One way to improve the efficacy in eradicating the infection is to deliver the antifungal locally in the oral cavity. Better stability and longer residence time will allow more of the antifungal to penetrate through the oral mucous layer to act on *Candida* species for longer duration of time. Therefore some researchers had prepared and reported new formulation such as gels, mucoadhesive tablets, pH sensitive excipients composition mucoadhesive microspheres, which were able to reside in oral cavity for an extended period for more effective candidiasis eradication[3,4]. As conventional drug delivery system does not remain in the oral cavity for prolonged period, they are unable to deliver the antifungal agents to the site of infection in effective concentrations and in fully active forms. Hence attention has been focused on extended release topical antifungal formulations for prolonging active drug concentrations in the oral cavity. Jung *et al*.[3] developed mucoadhesive thermo-sensitive gels and found that *in vivo* antifungal activity of clotrimazole (CT) was significantly prolonged. Ning *et al*.[4] formulated CT containing vaginal proliposomes, which showed prolonged drug release and may increase amount of drug retention into the mucosa to result in more antifungal efficacy. Using suitable carriers which can effectively administer the drug for an extended period of time not only reduces the systemic side effects but also improves the therapeutic efficacy and patient compliance.

The aim of the present work was to develop pH triggered system using carbopol and ion triggered using gellan gum, *in situ* gels for local release of CT, in the buccal cavity for the treatment of oropharyngeal candidiasis. A combination of carbopol - hydroxypropylmethylcellulose (HPMC) and gellan gum-HPMC were investigated as vehicle for the formulation.

## MATERIALS AND METHODS

Clotrimazole was a gift sample from Bayer Pharmaceuticals Private Limited, Thane, India. Carbopol-934P (CP-934P) and hydroxypropylmethylcellulose (HPMC E50LV) were obtained as gift samples from Goodrich and Colorcon Asia Pvt. Ltd, Mumbai, India. Gellan gum was procured from Hi-Media Pvt Ltd, Mumbai, India. All other materials used were of analytical grade. Subcultures of *Candida albicans* was obtained from MTCC (Microbial Type Culture Collection), Chandigarh, India.

### Preparation of *in situ* gelling systems:

Aqueous solutions of varying concentration containing Carbopol 934P and HPMC (E50LV) were prepared and evaluated for gelling capacity and viscosity in order to identify the composition suitable for as *in situ* gelling systems[5] (Formulation codes CF1, CF2, CF3, CF4, CF5 and CF6). Many experiments were conducted by varying the concentration of these polymers in order to identify the optimum concentration required for the gel forming solution. Dispersion containing carbopol 934P of different concentration (0.2-1.4% w/v) were initially prepared in pH 4.5 phosphate buffer solutions.

Gellan gum solutions of various concentrations were prepared by adding the gum to deionised water containing 0.17% w/v sodium citrate and heated to 90° while stirring. After cooling to below 40° appropriate amounts of calcium chloride (0.05% w/v) was added into the sol (formulation codes GF1, GF2, GF3, GF4, GF5, GF6). CT was dissolved in ethanol (2% w/w) and was added to the solution. The mixture was stirred by using a magnetic stirrer to ensure thorough mixing. The pH of the gel (1 g) was determined using a calibrated pH meter (Elico, Hyderabad, India). The values were taken as average of 3 reading.

### Determination of mucoadhesive force:

The mucoadhesive force of *in situ* gels on tissue specimen (cheek of chicken) was determined by means of mucoadhesive force measuring apparatus, fabricated in our laboratory. The pieces of tissue were stored frozen in phosphate buffer at pH 7.4, and thawed to the room temperature before use. At the time of testing, a section of tissue was secured (keeping the mucosal side out) to the upper side of a glass vial using a cyanoacrylate adhesive. The diameter of each exposed mucosal membrane was 1.5 cm. The vials were equilibrated and maintained at 37° for 10 min. One vial with a section of tissue was connected to the balance and the other vial was fixed on a height adjustable pan. To the exposed surface of the tissue attached on the vial, a constant amount of 0.1 g gel was applied. Before applying the gel, 150 μl of simulated saliva solution (2.38 g NaHPO_4_, 0.19 g KH_2_PO_4_ and 8 g NaCl in 1000 ml of distilled water adjusted to pH 6.75) was evenly spread on the surface of the test membrane. The height of the vial was adjusted such that the gel could adhere to the mucosal surface of both vials. Immediately, a constant force of 0.5 N (Newton) for 2 min was applied to ensure intimate contact between the tissue and the sample. The upper vial was then moved upwards at a constant force, while it was connected to the balance. Weights were added at a constant rate to the pan on the other side of the modified balance until the two vials were separated[5]. The bioadhesive force, expressed as the detachment stress in dynes/cm^2^, was determined from the minimal weights needed to detach the tissues from the surface of each formulation, using the following Eqn[6]. Detachment stress (dynes/cm^2^) = (m×g)/a, where ‘m’ is the weight added to the balance in grams; ‘g’ is the acceleration due to gravity taken as 980 cm/s^2^; and ‘a’ is the area of tissue exposed. Effect of varying contact time (1, 2, 3, 5 and 10 min) was investigated for some of the gel preparations to optimize initial contact time. In brief, formulations were allowed to be in contact with mucosa for carrying contact time (1, 2, 3, 5, and 10 min.), and the bioadhesive force was determined as discussed above[7]. Contact time that resulted in maximum bioadhesive strength was selected as optimum contact time required for adequate adhesion. All the above mentioned experiments were carried out in triplicates.

### Measurement of viscosity of sols:

Viscosity determinations of the prepared *in situ* gels as well as sols were carried out on a cone and plate geometry viscometer (Brookfield, Massachusetts, USA), using spindle No 40. Viscosity of *in situ* gelling solutions was measured at different angular velocities at a temperature of 37°. A typical run comprised changing of the angular velocity from 0.0 to 100 rpm[6]. The averages of two readings were used to calculate the viscosity. Evaluations were conducted in triplicate.

### Antifungal efficacy studies:

The antifungal efficacy study against *Candida albicans* was determined by agar diffusion method employing ‘cup plate technique’. Sterile solutions of CT in DMSO (standard solution) and the developed gel having the pH adjusted to 7.0, were poured into cups (0.1 ml of 0.1% w/v) bored into sterile malt yeast agar previously seeded with test organism. After allowing diffusion of the solutions for 2 h, the agar plates were incubated at 37° for 48 h. The zone of inhibition (ZOI) was measured and compared with that of pure drug. The entire operation was carried out in aseptic condition through out the study. Each solution was tested in triplicate. Both positive and negative controls were maintained throughout the study[8].

### Determination of gel strength:

The method by which the properties of polymeric system may be conveniently determined is texture profile analysis. A TA-XT2 Texture analyzer (The experiments were conducted at Digital Scientific Equipments, RK Puram, New Delhi). The experiment was done by placing the gels in standard beaker below the probe. In this an analytical probe is then immersed into the sample. The Texture Analyzer was set to the ‘gelling strength test’ mode or compression mode with a test-speed of 1.0 mm/s. An acquisition rate of 50 points per seconds and a trigger force of 5 g were selected. An aluminum probe of 7.6 cm diameter was used for all the samples. The study was carried out at room temperature[9]. The force required to penetrate the gel was measured as gel strength in terms of g.

### Gelling capacity:

The gelling capacity was determined by placing a drop of the system in a vial containing 2 ml of simulated salivary fluid (pH 6.8) freshly prepared and equilibrated at 37° and visually assessing the gel formation and noting the time for gelation and the time taken for the gel formed to dissolve. Different grades were allotted as per the gel integrity, weight and rate of formation of gel with respect to time.

### Spreadability:

For the determination of spreadability, excess of sample was applied between the two glass slides and was compressed to uniform thickness by placing 1000 g weight for 5 min. Weight (50 g) was added to the pan. The time required to separate the two slides, i.e. the time in which the upper glass slide moves over the lower plate was taken as measure of spreadability (S)^7^. S=M×L/T, where M = weight tide to upper slide, L = length moved on the glass slide, T = time taken.

### *In situ* release studies:

For carrying out *in situ* release studies and determination of duration of bioadhesion/erosion, a flow through apparatus[4] was designed based on the modification of a “flow through device cell”. The flow through cell was made of glass and had a length of 10.5 cm and a diameter of 2.1 cm. It was closed at one end and open at the other. In the center of the lower base there was a cavity of 1.6 cm length and 1.5 cm depth for placement of the chicken cheek mucous membrane. Dissolution medium pH 6.8 (simulated salivary pH) was pumped at a flow rate of 0.6 ml/min (corresponding to mean resting salivary flow rate) using flow regulators. Sols were added from the top so that upon contact with the salivary fluid get converted to gel form. The gels settled on the mucous membrane, exhibited mucoadhesive property. Samples (2 ml) were withdrawn at different time intervals from the reservoir till the gel was completely eroded. The cumulative percent drug release was determined by measuring the absorbance at 260 nm.

### Statistical analysis

The results obtained from the experiments of mucoadhesive strength and release studies were analyzed statistically using multivariate tests. A statistically significance difference was conducted using SPSS version 13 and difference was considered to be significant at *p*<0.05.

## RESULTS AND DISCUSSION

The formulations of this study contained Ca^++^ ions in complexed form and the release of which in the slightly acidic conditions of the buccal cavity ensured reproducible gelation of the gellan gum. The quantities of the complexing agents calcium chloride and sodium citrate must be such that there is no free calcium in free ionic form in the formulation so as to ensure that they are in fluid state before administration, but sufficient Ca^++^ ions must be released when the complex is broken down (due to dissociation) in presence of simulated salivary fluid to cause gelation. Determination of the optimum amounts of complexing agents for gellan gum sols (Table 1) showed that only those containing 0.05% (w/v) calcium chloride in combination with 0.17% (w/v) sodium citrate were found to be satisfactory to cause gelation. Low level of cations present in the solution was sufficient to hold the molecular chains together and inhibit hydration. As a result the experiment was performed with formulations containing 0.05% (w/v) calcium chloride and 0.17% (w/v) sodium citrate. The use of carbopol for *in situ* gel forming systems is substantiated by the property of its aqueous solutions into stiff gels, when the pH is raised. However, the concentrations of carbopol required to form stiff gels prepared in acidic solutions (pH 4.5) are not easily neutralized by the buffering action of the salivary fluid (pH 6.8).

**TABLE 1:** COMPOSITION OF VARIOUS FORMULATIONS USED IN THE PREPARED *IN SITU* GELS

Formulation	Clotrimazole (%)	Gellan gum (%)	Carbopol (%)	HPMC (%)	Sodium citrate (%)	Calcium chloride (%)	Deionized water (%) up to
GF1	0.1	0.1	-	0.5	0.17	0.05	10
GF2	0.1	0.2	-	0.5	0.17	0.05	10
GF3	0.1	0.3	-	0.5	0.17	0.05	10
GF4	0.1	0.4	-	0.5	0.17	0.05	10
GF5	0.1	0.5	-	0.5	0.17	0.05	10
GF6	0.1	0.75	-	0.5	0.17	0.05	10
CF1	0.1	-	0.2	0.5	0.17	0.05	10
CF2	0.1	-	0.4	0.5	0.17	0.05	10
CF3	0.1	-	0.6	0.5	0.17	0.05	10
CF4	0.1	-	0.8	0.5	0.17	0.05	10
CF5	0.1	-	1.2	0.5	0.17	0.05	10
CF6	0.1	-	1.4	0.5	0.17	0.05	10

A reduction in the concentration of primary polymers (gellan gum/carbopol) without compromising the gelling capacity and rheological properties of the delivery system may be achieved by the addition of viscosity enhancing polymers such as HPMC. The HPMC also helped the gel to rapidly settle due to its higher density than the gels prepared without HPMC. This also helped the gels for its adhesion property to the mucous membrane and subsequent prolonged release. Aqueous sols exhibited pH values in range of 4.0 to 7.5, at 25° (Table 2). The pH of all the formulations was adjusted to 6.5-7 with triethanolamine.

**TABLE 2:** CHARACTERISTICS OF VARIOUS CLOTRIMAZOLE GEL FORMULATIONS

Formulations	pH*	Viscosity* (cps)	Spreadbility g.cm/s	Drug Content (%w/w)	*Muco- adhesive force dynes/cm^2^	Gelling Capacity	Gel strength g/s
GF1	7.2	11000	25.2	98.7	52.2±2.4	−	0.5
GF2	7.1	11200	25.8	98.6	53.5±1.09	+	2
GF3	7.2	12020	26	98.8	48.4±3.4	+	3.5
GF4	7.3	12540	27.5	98.5	51.5±5.3	++	4
GF5	7.2	14000	28.5	90.4	52.5±7.5	+++	6.5
GF6	7.5	16000	30.7	98.5	54±5.6	+++	7.2
CF1	6.8	600	10.12	95.3	30.6±0.7	−	0
CF2	6.2	700	11.01	99.9	32.7±2.8	−	0.5
CF3	6.4	850	11.5	98.8	36.5±1.5	+	1.2
CF4	6.4	1050	11.8	98.3	48±1.2	+	1.5
CF5	6.2	1234	12.2	99.7	52.5±2.8	++	2
CF6	6.1	1533	12.4	99.4	55.6±4.2	++	2.5

* Average of three reading;

− No gelation;

+ Gel after few minutes, dissolved rapidly;

++ Gelation immediately, remains for few hours;

+++ Gelation immediately, remains for extended period.

**Fig. 1:**
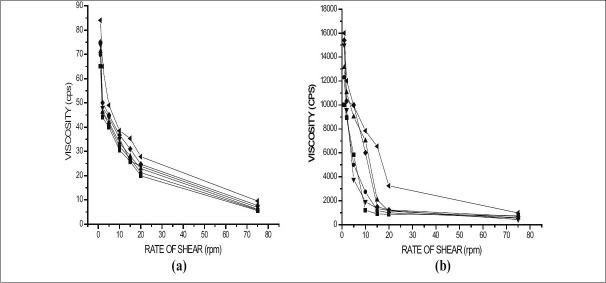
Viscosity of the various formulations using gellan gum (a) Various sols represented by (–▲–) Formulation GF1, (–▼–) Formulation GF2, (–▪–) Formulation GF3, (–●–) Formulation GF4, (–♦–) Formulation GF5, (–◀–) Formulation GF6, and (b) Various Gels represented by (–▲–) Formulation GF1, (–▼–) Formulation GF2, (–▪–) Formulation GF3, (–●–) Formulation GF4, (–♦–) Formulation GF5, (–◀–) Formulation GF6.

**Fig. 2:**
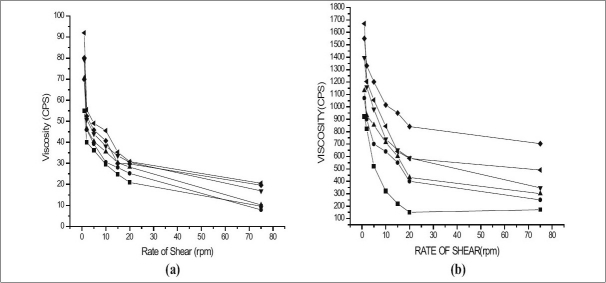
Viscosity of the various formulations using carbopol (a) Various sols represented by (–▲–) Formulation CF1, (–▼–) Formulation CF2, (–▪–) Formulation CF3, (–●–) Formulation CF4, (–♦–) Formulation CF5, (–◀–) Formulation CF6, and (b) Various Gels represented by (–▲–) Formulation CF1, (–▼–) Formulation CF2, (–▪–) Formulation CF3, (–●–) Formulation CF4, (–♦–) Formulation CF5, (–◀–) Formulation CF6.

The two main prerequisites of an *in* situ gelling system are viscosity and gelling capacity. To instill easily at the affected site the formulation must possess optimum viscosity. Further, the formulation should undergo rapid sol to gel transition upon contact at the affected site. Hence, the viscosity of sols and gels of various formulations was determined at various shear rates (figs. 1 and 2). It was found that as the shear rate increased the viscosity of gel decreased. Also, the increase in polymer concentration caused increase in viscosity of the formed gel. These findings indicated the differences between viscosity of gels and their corresponding sols. The formulation GF6 having the maximum concentration of gellan gum showed maximum viscosity for both sols and its corresponding gels. It was also found that higher content of gellan gum in the formulation (GF6), not ideal for *in situ* gel formulation, since such formulation exhibits higher viscosity for sols, hence not pourable. In comparison, formulation containing carbopol 934P showed no remarkable sol to gel conversion. This may be attributed to the lower pH of simulated salivary fluid (pH 6.8) as discussed earlier. It has been showed by Srividya *et al*, that carbopol could show better sol to gel conversion at pH 7.2 and higher[10].

All the formulations exhibited shear thinning pseudo-plastic behavior with thixotrophy. Further, it was also found that, all the formulations were liquid at room temperature and underwent rapid gelation when the pH was raised to 6.8. The viscosity of formulation contributed to the product adhesiveness, reflecting the importance of product rheology on this parameter[11].

The mucoadhesive force is an important physicochemical parameter for topical application in buccal cavity. The effect of different concentrations of CT gel formulation on mucoadhesive force is shown in Table 2. The bioadhesive force was significantly increased as the concentration of mucoadhesive polymer increased in the range of 0.6-3.5% (*p*<0.05). Formulation GF6 (containing the maximum polymer ratio 0.75:0.5) using gellan gum and CF5, CF6 containing carbopol, exhibited maximum mucoadhesive strength. This also proved that carbopol (up to 55 dyes/cm^2^) has better mucoadhesive property than gellan gum (up to 54 dynes/cm^2^), even though, they showed poor gelling capacity. The results also indicated that the presence of secondary polymer (HPMC) significantly increased the viscosity as well as the mucoadhesive property.

**Fig. 3:**
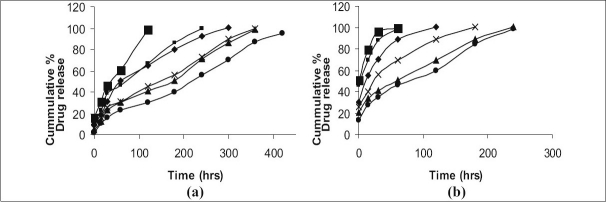
The *in vitro* release profiles of clotrimazole *in situ* gels (a) Gels prepared using Gellan gum, (–▪–) Formulation GF1, (–▪–) Formulation GF2, (–♦–) Formulation GF3, (–χ–) Formulation GF4, (–▲–) Formulation GF5, (–●–) Formulation GF6, and (b) Gels prepared using Carbopol (–▪–) Formulation CF1, (–▪–) Formulation CF2, (–♦–) Formulation CF3, (–χ–) Formulation CF4, (–▲–) Formulation CF5, (–●–) Formulation CF6,

The *in vitro* dissolution profile of CT from the gels containing different concentration of gellan gum and carbopol based gels is shown in fig. 3. The release of drug from these gels was characterized by an initial phase of high release (burst effect) and as the gelation proceeded, the remaining drug was released at a slower rate (second phase). This bi phasic pattern of release is a characteristic feature of matrix diffusion kinetics. The initial burst effect was considerably reduced with increase in polymer concentration. The formulation GF1and GF2 containing the lower polymer ratio (0.1: 0.5 and 0.2: 0.5) showed the release profile only up to 4 h, whereas formulation having higher polymer ratio i.e., GF6, showed only 50% release at the end of 6 h. Since we were inclined to formulate *in situ* gel which show 90% release profile within 6 h, GF1, GF2 and GF6 formulations were not found to be ideal formulations for *in situ* gels. In comparison, the gels containing carbopol having the maximum concentration CF6 could show release only up to 5 h, though, the other formulations of carbopol disintegrated rapidly and released the drug within 4 h. However, these findings clearly showed that the gels have the ability to retain CT at higher concentration of carbopol (CF6) and premature release of drug can be avoided. Hence GF4, GF5 and CF6 formulations were chosen to meet the above said criterion.

The examination of the correlation coefficient ‘r’ indicated that the drug release followed diffusion controlled mechanism from the *in situ* gels, as the values of ‘r’ for first order (ranged from 0.9183 to 0.9891) found to be higher in comparison to zero order (ranged from 0.6629 to 0.9333) and Higuchi's square root of time (ranged from 0.6558 to 0.9708). It was understood to be predominant first order release pattern. Further, to understand the drug release mechanism, the data were fitted into Peppas exponential model M^t^/M^∞^=Kt^n^, where M^t^/M^∞^ is the fraction of drug released after time ‘t’ and ‘K’ is kinetic constant and ‘n’ is release exponent which characterizes the drug transport mechanism[12]. The values ‘n’ were in the range of 0.3224–0.428, which was further indicative of the drug release following diffusion first order release kinetic mechanism (Table 3).

**TABLE 3:** RELEASE EXPONENT VALUES AND RELEASE RATE CONSTANT VALUES FOR DIFFERENT FORMULATION

Formulation Code	Kinetic Models				
	
	Zero Order	First Order	Higuchi	Korsmeyer et al.,	
				
	r	r	r	r	r
GF1	0.8011	0.9872	0.7764	0.4431	0.9863
GF2	0.8273	0.9654	0.8442	0.3224	0.8942
GF3	0.9850	0.9354	0.9708	0.4280	0.9537
GF4	0.6629	0.9183	0.6220	0.3588	0.9912
GF5	0.9333	0.9816	0.8750	0.3915	0.9152
GF6	0.7825	0.9891	0.6558	0.4280	0.9239
CF1	0.4616	0.9771	0.8876	0.4330	0.9368
CF2	0.5326	0.8764	0.8845	0.5140	0.8302
CF3	0.5534	0.7653	0.8867	0.4670	0.9897
CF4	0.6577	0.8836	0.8621	0.4850	0.9902
CF5	0.7673	0.9916	0.7633	0.4430	0.9852
CF6	0.8821	0.9991	0.7431	0.4810	0.9939

The antifungal activity of the tested *in situ* formulations was found to be equal to that of the reference standard. It was observed that the plain formulation used in the study exhibited no antifungal activity. The students ‘t’ test showed that there is no significant difference in the zone of inhibition with the gel formulation in comparison to the reference standard at P <0.05.

Gel formulation of CT with mucoadhesive properties was found to be promising for prolonging buccal residence time and thereby better therapeutic effects. In addition, they provide intimate contact between a dosage form and the absorbing tissue which may result in high drug concentration in local area. The *in situ* formulation may improve the patient acceptability, as the formulation is applied in the form of sols, which upon contact forms the corresponding gels causing less irritation or pain.

## References

[1] Melo NR, Taguchi H, Culhari VP, Kamei K, Mikami Y, Smith SN (2009). Oral candidiasis of HIV-infected children undergoing sequential HIV therapies. Med Mycol.

[2] Gallardo JM (2008). Xerostomia: Etiology, diagnosis and treatment. Rev Med Inst Mex Seguro Soc.

[3] Chang JY, Oh YK, Kong HS, Kim EJ, Jang DD, Nam KT (2002). Prolonged antifungal effects of Clotrimazole-containing mucoadhesive thermosensitive gels on vaginitis. J Control Release.

[4] Ning MY, Guo YZ, Pan HZ, Yu HM, Gu ZW (2005). Preparation and evaluation of proliposomes containing clotrimazole. Chem Pharm Bull (Tokyo).

[5] Wu C, Qi H, Chen W, Huang C, Su C, Li W (2007). Preparation and evaluation of a Carbopol/HPMC-based in situ gelling ophthalmic system for puerarin. Yakugaku Zasshi.

[6] Ikanth PS, Mishra B (2008). Floating *in situ* gelling system for stomach site-specific delivery of clarithromycin to eradicate *H. pylori*. J Control Release.

[7] Mitra J, Mohammad RR, Hedayte S (2006). Mucoadhesive and drug release properties of benzocaine gel. Iranian J Pharm Sci.

[8] El laithy HM, El-Shaboury KM (2002). The development of cutina lipogels and gel microemulsion for topical administration of fluconazole. AAPS PharmSciTech.

[9] Walewijk A, Cooper White JJ, Dunstan DE (2008). Adhesion measurements between alginate gel surfaces via texture analysis. Food Hydrocolloids.

[10] Srividya B, Cardoza RM, Amin PD (2001). Sustained ophthalmic delivery of ofloxacin from a pH triggered in situ gelling system. J Control Release.

[11] Hongyi Q, Wenwen C, Chunyan H, Chuming C, Wenmin L, Chunjie W (2007). Development of a poloxamer analogs/carbopol-based *in situ* gelling and mucoadhesive ophthalmic delivery system for puerarin. Int J Pharm.

[12] Raghuram Reddy K, Srinivas M, Srinivas R (2003). Once-daily sustained-release matrix tablets of nicorandil: Formulation and *in vitro* evaluation. AAPS PharmSciTech.

